# Trauma and identity: a narrative study among refugee torture survivors

**DOI:** 10.3402/ejpt.v4i0.22987

**Published:** 2013-12-05

## XIII ESTSS Conference: “Trauma and its clinical pathways: PTSD and beyond”, Bologna, June 2013 POSTER, JUNE 8

## The Spectrum of Trauma-Related Disorders

### Trauma and identity: a narrative study among refugee torture survivors

#### Adrienn Kroó Department of Psychology, University of Pécs, Pécs, Hungary; Cordelia Foundation for the Rehabilitation of Torture Victims, Budapest, Hungary

The classical concept of PTSD has been criticized for many reasons, including its cultural limitations and negligence of long-lasting changes to personality, which has been documented in cases of chronic interpersonal trauma. The concept of complex PTSD has addressed these shortcomings and specifies “alterations in self-perception” as one of the characteristic changes in functioning following extreme and chronic trauma. Torture survivors and refugees are a population particularly affected by cumulative traumatization. The experience of torture comprises the deliberate and systematic devastation of values, beliefs, self-concept, and personality development. It has severe effects on the survivors’ fundamental trust, self-identity, and attachment. Meanwhile, refugee trauma involves the long-term experience of discrimination, persecution, helplessness, and humiliation, which also has a significant impact on the individual's personal and collective identity. A number of studies and theories now exist, which describe the psychological effect of torture and refugee trauma on identity. However, the issue of torture and life in exile is generally discussed separately. The present research aims at integrating the two phenomena and focuses on the unique and combined effect that torture and refugee trauma has on the survivors’ identity. The qualitative study comprises narrative in-depth interviews with refugee torture survivors and applies a psychoanalytic framework for interpreting the data.

